# Predictive Validity of the Snatch Pull Force-Velocity Profile to Determine the Snatch One Repetition-Maximum in Male and Female Elite Weightlifters

**DOI:** 10.3390/jfmk6020035

**Published:** 2021-04-16

**Authors:** Ingo Sandau, Helmi Chaabene, Urs Granacher

**Affiliations:** 1 Research Group Weightlifting, Institute for Applied Training Science, D-04109 Leipzig, Germany; 2 Division of Training and Movement Sciences, University of Potsdam, Research Focus Cognition Sciences, D-14469 Potsdam, Germany; chaabanehelmi@hotmail.fr (H.C.); urs.granacher@uni-potsdam.de (U.G.)

**Keywords:** validation study, performance, monitoring, training

## Abstract

Background: The prediction of one repetition-maximum (1RM) performance from specific tests is highly relevant for the monitoring of training in weightlifting. Therefore, this study aimed at examining the predictive validity of the theoretical 1RM snatch (*snatch_th_*) computed from the two-point snatch pull force-velocity relationship (FvR_2_) to determine actual snatch 1RM performance in elite weightlifters. Methods: Eight (three female, five male) elite weightlifters carried out a 1RM snatch test followed by a snatch pull test with loads of 80% and 110% of the previously determined 1RM snatch. Barbell kinematics were determined for all lifts using video-tracking. From the snatch pull barbell kinematics, the snatch pull FvR_2_ was modeled and the *snatch_th_* was calculated. Results: The main findings indicated a non-significant (*p* = 0.706) and trivial (*d* = 0.01) mean difference between the actual 1RM snatch performance and the *snatch_th_*. Both measures showed an extremely large correlation (*r* = 0.99). The prediction accuracy of the actual 1RM snatch from *snatch_th_* was 0.2 ± 1.5 kg (systematic bias ± standard deviation of differences). Conclusions: This study provides a new approach to estimate 1RM snatch performance in elite weightlifters using the snatch pull FvR_2_. The results demonstrate that the *snatch_th_*-model accurately predicts 1RM snatch performance.

## 1. Introduction

Systematic performance testing (i.e., monitoring) is a cornerstone to detect intraindividual changes over time for general fitness (e.g., muscle strength, power) and sport-specific performance parameters in elite weightlifters [1]. A widely used and highly reliable test in weightlifting is the one-repetition maximum (1RM) test, for instance, during the snatch [2]. In addition to the 1RM test, performance assessment in weightlifting included vertical jump tests, isometric mid-thigh pull tests, and back/leg extensor tests [3,4,5].

A prerequisite of a test to be included for performance assessment is that it sufficiently complies with psychometric properties such as validity, reliability, and responsiveness [6]. In terms of test validity, predictive validity is highly relevant for elite sports [7]. In this context, Joffe and Tallent [8] examined the predictive validity of peak power during vertical jumping and peak force during the isometric mid-thigh pull using a multiple linear regression model to determine weightlifting performance (i.e., 1RM snatch, clean and jerk, and total) in highly trained female weightlifters aged 23 years. These authors reported high relative (R^2^ = 0.94–0.95) and absolute (standard error of estimate (SEE) = 3.8–9.5 kg) predictive validity. In general, a multiple linear regression model reflects the statistical association between independent (e.g., peak power during vertical jumping, peak force during isometric mid-thigh pull peak force) and dependent (e.g., 1RM snatch) variables in a given sample (i.e., group data). Of note, results from regression models are always specific to the population under investigation [9]. Accordingly, a low predictive value of an estimated outcome can be obtained if established regression equations are translated to different samples (i.e., shrinkage) [10]. This is even more prevalent if the prediction equation has been derived from a rather small sample [10]. In this context, there is preliminary evidence that the prediction of individual performance levels using an individualized biomechanical approach, rather than statistical, provides higher external validity [9].

The individual load-velocity relationship (LvR) (i.e., linear regression model of barbell load vs. barbell velocity) is a good example of such a widely applied biomechanical approach that has the potential to predict 1RM during various strength exercises (e.g., bench press, back squat, deadlift, power clean) [11,12,13]. In this easy-to-administer approach, the estimated 1RM is the intercept of the extrapolated LvR-regression-line with the previously determined minimal velocity threshold of a maximal lift [11]. In addition to the LvR, the force–velocity relationship (FvR) represents another method to predict 1RM performance [14,15]. A previous study used a commercially available linear encoder (Musclelab, Ergotest, Norway) to compute 1RM bench press performance (systematic bias ± standard deviation of differences (SDD) = 5.4 ± 5.7 kg) from FvR in strength trained adults aged 29 years [15]. However, these authors left the underlying computational algorithm in a black box. In another study, a combined LvR and FvR approach was used to predict 1RM chest press (systematic bias ± SDD = −1.3 ± 1.2 kg) and leg press performance (systematic bias ± SDD = −1.8 ± 2.1 kg) in recreationally active participants aged 24 years [14]. Recently, Sandau, et al. [16] introduced a conceptual biomechanical model to compute the (theoretical) 1RM snatch performance (i.e., *snatch_th_*) from the two-point snatch pull FvR (FvR_2_) in elite male weightlifters aged 27 years. Results indicated high reliability of the *snatch_th_* (percentage standard error of measurement (SEM%) = 0.71%, intraclass correlation coefficient (ICC) = 0.99) [16]. However, the predictive validity of the *snatch_th_* to determine actual 1RM snatch performance has not yet been investigated.

To the best of the authors’ knowledge, no study has examined the accuracy to predict 1RM performance using FvR-profiling with specific weightlifting exercises (i.e., snatch, clean and jerk). Therefore, this study aimed at examining the predictive validity of the *snatch_th_* computed from the snatch pull FvR_2_ to determine the actual snatch 1RM performance in elite male and female weightlifters. Based on the computational similarities of 1RM prediction from LvR with the *snatch_th_* from FvR_2_ [16], we hypothesized that the *snatch_th_* can be used to accurately predict actual 1RM snatch performance in elite weightlifters.

## 2. Materials and Methods

### 2.1. Subjects

Eight elite weightlifters (male = 5; female = 3; age range: 18–29 years), all members of the German national team, volunteered to participate in this study (Table 1). At the time of the study, all weightlifters regularly competed at national and international events and had >6 years of systematic weightlifting training. They were free from any musculoskeletal or neurological diseases or injuries at the time of data collection. This study was conducted according to the latest version of the Declaration of Helsinki, and the experimental protocol was approved by the local Ethics Board of the Institute for Applied Training Science (approval number: ER_2020.28.09_4).

### 2.2. Data Collection

Data were collected during the preparation period of a macrocycle. Testing was performed on a regular training day at the beginning of the weightlifting training session. Before the tests, an individualized warm-up program was conducted for 15–20 min including cycling on an ergometer at submaximal intensity, mobility exercises with and without the barbell. After the warm-up, the participating weightlifters started with a 1RM snatch test. They were encouraged to reach their maximal snatch performance (i.e., 100% = actual 1RM) using 6–8 loads with 1–2 repetitions per load condition. The rest between load conditions and repetitions was 3 and 2 min, respectively. Weightlifters were instructed to perform every lift at maximal effort. After the 1RM snatch test, a 5 min rest was implemented before the snatch pull test was conducted. During the snatch pull test, weightlifters were encouraged to lift two consecutive repetitions at 80% and 110% of the previously determined 1RM snatch at maximal effort and starting with the 110% load condition. The rest between load conditions was 3 min and between repetitions 2 min. Lifting straps were used during all snatch pull trials.

The snatch and the snatch pull lifts were video recorded (Canon, Legria HF G26) and analyzed using a custom-made real-time barbell tracking software (Realanalyzer, IAT, Leipzig, Germany) [17]. The position of the digital camera followed a routine set-up, with the camera placed 1 m above the floor and positioned next to the athlete at a distance of 5 m. The vertical barbell velocity was computed as the 1st derivative of vertical barbell position data. This system demonstrated excellent absolute (SEM%) and relative (ICC) test-retest reliability for the measurement of maximal barbell velocity (SEM% = 0.72%, ICC > 0.99) and maximal distance (SEM% = 0.45%, ICC > 0.99) [18].

### 2.3. Data Processing

**Figure 1:**
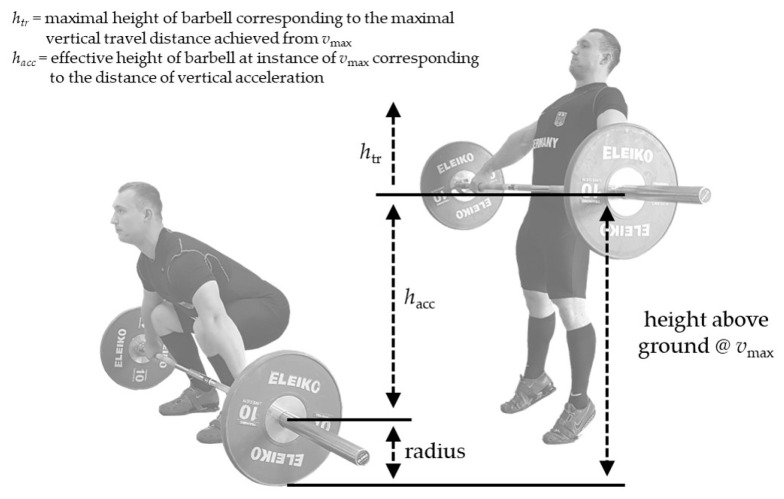
Schematic illustration of the snatch pull exercise from start position (**left**) to position of maximal vertical barbell velocity (*v_max_*) (**right**) with vertical barbell kinematic parameters.

Maximal vertical barbell velocity was measured during the 1RM snatch (denoted as *v_thres_*) and during all snatch pull trials (denoted as *v_max_*). If *v_max_* values from two consecutive snatch pull lifts differed more than 0.1 m·s^−1^ at a specific load condition, a third repetition was conducted. In addition, the distance of vertical acceleration (*h_acc_*, i.e., vertical position of the barbell at the instant of *v_max_* minus the radius of the barbell plates (0.225 m)) was taken from the measurements during the snatch pull at the 110% load (Figure 1).

Averaged values from two consecutive snatch pull lifts for *v_max_* (80% and 110% condition) and *h_acc_* (110% condition) were used for further analyses. The snatch pull FvR_2_ was modeled using linear regression with mean vertical barbell force ($\bar{F}$) and velocity ($\bar{v}$) from the 80% and 110% load conditions as input parameters. With reference to the approach presented by Samozino et al. [19], the mean vertical barbell force and velocity were computed from vertical barbell kinematics (i.e., *v_max_*, *h_acc_*).

The mean vertical barbell velocity was computed as follows:

(1) $$\bar{v}=\sqrt{\frac{g\times h_{tr}}{2}}$$

In this equation, *h_tr_* stands for the maximal vertical travel distance of the barbell that is achieved from *v_max_*, and *g* stands for the gravitational acceleration:

(2) $$h_{tr}=\frac{v_{max}{}^{2}}{2g}$$

The mean external vertical force to accelerate the barbell was computed from *h_acc_*, *h_tr_*, and *m* (barbell mass) as follows:

(3) $$\bar{F}=m\times g\left(\frac{h_{tr}}{h_{acc}}+1\right)$$

From the snatch pull FvR_2_ regression model, the theoretical maximal mean vertical barbell velocity at zero barbell force (i.e., ${\bar{v}}_{0}$; intercept of velocity axis), the theoretical maximal mean vertical barbell force at zero barbell velocity (i.e., ${\bar{F}}_{0}$; intercept of force axis), and the theoretical maximal mean vertical barbell power (i.e., ${\bar{P}}_{max}$) were computed [20]. The ${\bar{v}}_{0}$, ${\bar{F}}_{0}$, and ${\bar{P}}_{max}$ were used as typical FvR parameters to evaluate the maximal mechanical capabilities of the neuromuscular system [21]. Based on the individual snatch pull FvR_2_ regression model, the *snatch_th_* was computed from barbell force at *v_thres_* of the 1RM snatch lift [16].

For this purpose, the mean vertical barbell force (${\bar{F}}_{thres}$) was computed for *v_thres_* from the snatch pull FvR_2_ regression model as follows:

(4) $${\bar{F}}_{thres}=\frac{{\bar{v}}_{thres}-{\bar{v}}_{0}}{slope_{FvR_{2}}}$$

where ${\bar{v}}_{thres}$ was computed from *v_thres_* (Equation (1)). The ${\bar{F}}_{thres}$ is the sum of the gravitational force due to barbell mass and the force from barbell acceleration. To obtain the barbell load at *v_thres_* (i.e., *snatch_th_*), ${\bar{F}}_{thres}$ has to be divided by the sum of *g* and the vertical barbell acceleration (*a_thres_*) to achieve *v_thres_*:

(5) $$snatch_{th}=\frac{{\bar{F}}_{thres}}{g+a_{thres}}$$

The vertical barbell acceleration to achieve *v_thres_* can be expressed as:

(6) $$a_{thres}=\frac{v_{thres}{}^{2}}{2\times h_{acc}}$$

To prove the predictive accuracy of the model, the computed *snatch_th_* was compared with the actual snatch performance of the 1RM test.

### 2.4. Statistical Analysis

The level of statistical significance was set for all tests at *p* ≤ 0.05. All statistical analyses were conducted using R (version 4.0.2). A list of the applied packages and functions can be found in the Supplementary Materials. The normal distribution of data was assessed and confirmed using the Shapiro–Wilk test. The absence of heteroscedasticity (i.e., the measurement error is related to the magnitude of the measured variable) of the measurements was confirmed using the Breusch–Pagan test. Therefore, no log-transformation of the raw data was necessary. The difference between the 1RM snatch and *snatch_th_* was analyzed using a paired-sample *t*-test alongside effect size (*d*) and 95% confidence limits (CL). The effect size was interpreted using conventions outlined by Hopkins [22] as small (|*d*| > 0.2), moderate (|*d*| > 0.6), large (|*d*| > 1.2), very large (|*d*| > 2.0), or extremely large (|*d*| > 4.0). An effect size <0.2 was deemed trivial. The correlation between the 1RM snatch and the *snatch_th_* was assessed using Pearson product-moment correlation coefficient (*r*) with 95% CL. Thresholds for the correlation coefficient were considered small (|*r*| > 0.1), medium (|*r*| > 0.3), large (|*r*| > 0.5), very large (|*r|* > 0.7), and extremely large (|*r*| > 0.9) [23]. The absolute and percentage measurement error was computed as SDD and SDD% with 95% CL. Additionally, the Bland–Altman analysis was used to compute the systematic bias (i.e., mean of measurements with 95% CL) and 95% limits of agreement (systematic bias ± 1.96 × SDD) with 95% CL. Significant systematic bias was prevalent if the range of the 95% CL of the mean difference did not contain the value 0. Furthermore, a Deming regression was performed to test for constant and proportional bias between the approaches. Significant constant bias was present if the range of the 95% CL of the intercept did not contain the value 0 and significant proportional bias was present if the range of the 95% CL of the slope did not contain the value 1 [24].

## 3. Results

Descriptive data are displayed in Table 2. The main finding indicated a non-significant (*p* = 0.706) and trivial (*d* = 0.01) mean difference between the actual 1RM snatch performance and the *snatch_th_* (Table 3).

**Figure 2:**
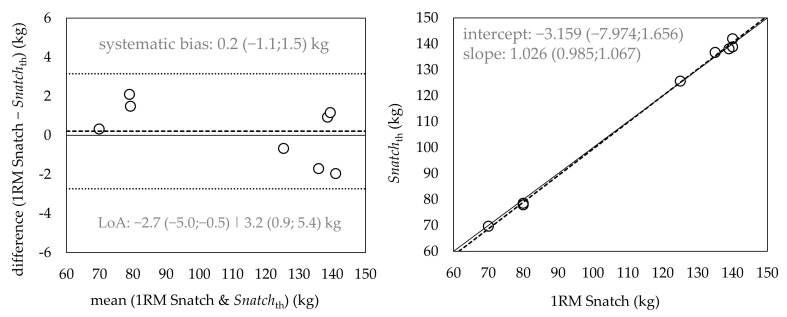
Results of the Bland–Altman analysis (**left**) and Deming regression (**right**) for the comparison between actual 1RM snatch and *snatch_th_*. The Deming regression plot illustrates the fitted linear model (dashed line) and the identity line (snatch 1RM = *snatch_th_*, slope = 1) (solid line). The Bland–Altman plot depicts mean differences between actual 1RM snatch and *snatch_th_* (dashed line) and 95% limits of agreement (dotted lines). Slope and intercept in the Deming regression plot are reported with 95% CL. The mean difference in the Bland–Altman plot is reported with 95% CL, and limits of agreement (LoA) are reported as systematic bias ± 1.96 × SDD with 95% CL. *Snatch_th_* = theoretical 1RM of the snatch computed from the snatch pull FvR_2_.

Results showed an extremely large correlation between the actual 1RM snatch and *snatch_th_* (*r* = 0.99) (Table 3). The outcomes of the Deming regression did not reveal a constant bias (i.e., CL of intercept contained the value 0) or proportional bias (i.e., CL of slope contained the value 1). In addition, the Bland–Altman analysis did not show any significant systematic bias (i.e., CL of the mean differences contained the value 0) (Figure 2). The SDD amounted to 1.5 kg, and the limits of agreement ranged from −2.7 to 3.2 kg.

## 4. Discussion

This study aimed to examine the predictive validity of the *snatch_th_* computed from the snatch pull FvR_2_ to determine the actual 1RM snatch performance of male and female elite weightlifters. It was hypothesized that the *snatch_th_* shows high accuracy to predict actual 1RM snatch performance in elite male and female weightlifters. Given that the main findings of this indicated high predictive validity of the *snatch_th_* to determine 1RM snatch performance in elite weightlifters, the study hypothesis was confirmed.

The ability of a testing protocol to accurately predict performance in the “real world” setting is a key aspect [7]. In this context, it has been shown that 1RM weightlifting performance can accurately be predicted for the snatch (SEE = 3.8 kg), clean and jerk (SEE = 6.0 kg), and total (SEE = 9.5 kg) using a multiple linear regression model with vertical jump peak power and isometric mid-thigh pull peak force data as predictors [8]. Besides statistical modeling, LvR-profiling is a widely accepted approach to predict 1RM performance that has, in fact, not yet been used for weightlifting competition exercises (i.e., snatch, clean and jerk). However, being similar to the snatch and clean, recent research was conducted on the prediction accuracy of 1RM in the power clean and deadlift exercise using individual LvR profiles [11,12,25]. For instance, Haff, Garcia-Ramos, and James [11] used the individual LvR to predict the 1RM power clean performance in recreationally trained males aged 26 years. These authors reported a measurement error (systematic bias ± SDD) of 1.4 ± 7.2 kg. Additionally, the individual LvR was used for the deadlift exercise to predict 1RM performance in resistance trained men aged 24 years [12,25]. Results indicated measurement errors (systematic bias ± SDD) of 0.6 ± 8.5 kg [25] and 0.7 ± 4.7 kg [12].

Of note, the prediction accuracy of the 1RM snatch from the snatch pull FvR_2_ model (i.e., *snatch_th_*) reported in the current study (systematic bias ± SDD = 0.2 ± 1.5 kg) is higher compared with the abovementioned studies that used either the multiple linear regression model [8] or the LvR-profiling [11,12,25]. The low measurement error of *snatch_th_* can be attributed to three primary reasons. First, the reliability of *snatch_th_* has been shown to be very high (SEM% = 0.71%, ICC = 0.99) [16]. This is due to the high reliability of the barbell tracking system used (i.e., Realanalyzer video analysis software) and to the high technical mastery of elite weightlifters. Second, *snatch_th_* was computed using the FvR_2_-profile of the snatch pull exercise (i.e., test exercise ≠ target exercise). During the snatch pull exercise, loads can be used that exceed the 1RM snatch load (i.e., overload) [26]. Therefore, the 1RM prediction using the snatch pull FvR_2_ linear regression model is an interpolation (i.e., 1RM condition is located within the measurements). In contrast, all LvR-based 1RM predictions were extrapolations (i.e., 1RM condition is located outside of measurements) given that the test exercise is the same as the target exercise for determining the 1RM. In fact, there is evidence that linear regression-based interpolations are less prone to measurement errors of independent variables than linear regression-based extrapolations [27]. Third, considering the inherent characteristics of the exercises, the snatch pull is a ballistic exercise (i.e., maximal effort lift to achieve maximal barbell velocity), while the deadlift is non-ballistic [28]. It has been shown during LvR-profiling that the reliability of peak and mean barbell velocity is lower in the bench press (i.e., non-ballistic) than in the bench press throw (i.e., ballistic), especially with light loads (e.g., 20% 1RM) [29]. The lower reliability of barbell velocity in a non-ballistic exercise will ultimately affect the slope of the LvR and the prediction of a 1RM load. In theory, the same principle can be applied for the non-ballistic deadlift, explaining the higher random error component (i.e., SDD) for 1RM prediction using LvR in the studies of Benavides-Ubric, Díez-Fernández, Rodríguez-Pérez, Ortega-Becerra and Pareja-Blanco [12] and Jukic, García-Ramos, Malecek, Omcirk and Tufano [25]. In contrast, although the power clean is considered a ballistic exercise [28], very light barbell loads may not be performed at maximal effort due to the catch phase of the power clean (i.e., barbell is “caught” on the shoulders). For the snatch pull, very light barbell loads may limit the lifters’ ability to generate maximal force output due to discomfort controlling the barbell at very high velocities [16]. In this case, the limited force output results in a submaximal barbell velocity output that influences the individual LvR slope and the accuracy of the 1RM prediction. In the study of Haff, Garcia-Ramos, and James [11], very light loads (i.e., starting at 30% of 1RM power clean with peak barbell velocities of 3.29 m·s^−1^) were used as initial load stages during LvR-profiling. For the same study, the aforementioned relation (i.e., submaximal barbell velocity output at very light loads) may explain the high random error component reported for the prediction of 1RM power clean using LvR-profiling.

This study presents some limitations that warrant discussion. First, the computed *snatch_th_* is based on the concept of threshold velocity (i.e., *v_thres_*) for a 1RM snatch [30]. This individual *v_thres_* needs to be precisely assessed during 1RM snatch lifts with the same measurement device as used during the snatch pull FvR_2_ testing. In addition, since barbell kinematics during weightlifting exercises can differ between barbell sides [17], all measurements need to be done at a standardized barbell side. Second, all lifts for the snatch pull FvR_2_ need to be executed at maximal effort. Any submaximal effort will affect the slope of the FvR_2_ and thus the model output (i.e., *snatch_th_*). Third, the size of the sample is rather small. However, the size of the overall population of elite weightlifters is small, resulting in a reduced sample size. Of note, the recruited sample is heterogeneous, including men and women with a wide range of 1RM snatch performances (70–140 kg). Such heterogeneity strengthens the external validity of the computed *snatch_th_* from the snatch pull FvR_2_. Finally, the presented approach is based on measurements from a custom-made video tracking software (i.e., Realanalyzer) with limited access. However, the computational base can also be used with other measurement units for the assessment of barbell kinematics. However, this would require another study to validate the accuracy of the *snatch_th_*-model using the new test set up.

## 5. Conclusions

In conclusion, this study provides a new approach to estimate 1RM snatch performance in elite weightlifters using the snatch pull FvR_2_ (i.e., *snatch_th_*). Our findings demonstrated that the *snatch_th_*-model accurately predicts 1RM snatch performance with a reduced random error of ±1.5 kg.

The findings of our study have high practical relevance for weightlifting in particular and strength and conditioning from a broader perspective. More specifically, the snatch pull FvR_2_ approach using 2D video analysis is an easy-to-administer test that can regularly be applied during weightlifting training as a valid alternative to the 1RM snatch test to assess individualized progression in weightlifting performance over time. However, when using the approach in practice, the abovementioned limitations need to be considered when interpreting the results. Given the comparable biomechanical structure of the snatch and the clean during the acceleration phase (i.e., pull), the current approach could be adopted for the clean pull exercise to predict the 1RM clean and/or 1RM power clean performance. However, this still needs to be confirmed in future studies.

## Figures and Tables

**Table 1:** Descriptive data in means and standard deviations of the study participants.

	Age (years)	Body Mass (kg)	1RM Snatch (kg)	1RM Clean and Jerk (kg)
Overall	23.3 ± 4.0	79.0 ± 16.6	124.8 ± 37.6	155.5 ± 41.7
Male	22.8 ± 3.3	87.4 ± 15.1	150.8 ± 11.5	184.2 ± 14.6
Female	24.0 ± 5.6	65.0 ± 6.0	81.3 ± 13.1	107.7 ± 12.9

**Table 2:** Descriptive data in means and standard deviations for the actual 1RM snatch, *snatch_th_*, snatch pull, and snatch pull FvR_2_.

1RM Snatch					*Snatch_th_*		
actual 1RM (kg)		*v_thre_* (m·s^−1^)			predicted 1RM (kg)		
113.6 ± 31.1		1.97 ± 0.12			113.4 ± 32.0		
**Snatch Pull**					**Snatch Pull FvR_2_**		
load @80% (kg)	load @110% (kg)	*v_max_* @80% (m·s^−1^)	*v_max_* @110% (m·s^−1^)	*h_acc_* @110% (m)	${\bar{v}}_{0}$ (m·s^−1^)	${\bar{F}}_{0}$ (N)	${\bar{P}}_{max}$ (W)
91.3 ± 26.1	123.5 ± 33.7	2.30 ± 0.14	1.83 ± 0.15	0.80 ± 0.08	2.20 ± 0.32	2569.4 ± 613.2	1408.7 ± 377.2

Notes: 1RM = one-repetition maximum of the snatch, *v_thres_* = maximal vertical barbell velocity of the 1RM snatch, *snatch_th_* = theoretical 1RM snatch computed from the two-point snatch pull force–velocity relationship (FvR_2_), load @80/110% = snatch pull barbell load relative to the 1RM snatch, *v_max_* = maximal vertical barbell velocity during the snatch pull, *h_acc_* = distance of vertical acceleration during the snatch pull, ${\bar{v}}_{0}$ = theoretical maximal mean vertical barbell velocity of snatch pull FvR_2_, ${\bar{F}}_{0}$ = theoretical maximal mean vertical barbell force of snatch pull FvR_2_, ${\bar{P}}_{max}$ = theoretical maximal mean vertical barbell power of snatch pull FvR_2_.

**Table 3:** Comparison between the actual 1RM snatch and the *snatch_th_* computed from the snatch pull FvR_2_.

*t*_(7)_	*p*	*d* (95% CL)	*r* (95% CL)	Diff (%)	SDD (95% CL) (kg)	SDD% (%)
0.393	0.706	0.01 (−0.02;0.04)	0.99 (0.99;1.00)	0.18	1.5 (1.0;3.1)	1.3

Notes: *t* = *t*-score from the paired-sample *t*-test, *p* = *p*-value from the paired-sample *t*-test, *d* = effect size, *r* = Pearson product-moment correlation coefficient, diff = percentage difference, SDD = standard deviation of differences, SDD% = percentage standard deviation of differences, 95% CL = 95% confidence limits.

## Data Availability

All underlying data are in the text, tables and figures.

## Supplementary Materials

The following are available online at https://www.mdpi.com/article/10.3390/jfmk6020035/s1, Document S1: Statistics_R.
