# Supplementary material for: Predictive Validity of the Snatch Pull Force-Velocity Profile to Determine the Snatch One Repetition-Maximum in Male and Female Elite Weightlifters
Source: J Funct Morphol Kinesiol. 2021 Apr 16;6(2):35. doi: 10.3390/jfmk6020035 (PMC8167790; doi:10.3390/jfmk6020035)
Supplement: Supplementary file 1 [file jfmk-06-00035-s001.zip › jfmk-1180583-supplementary.pdf]

Supplemental Material

Shapiro-Wilk test:

```
shapiro.test()
```

Breusch-Pagan test:

```
library(lmtest)
bptest()
```

Paired-sample t-test:

```
t.test()
```

Cohen d effect size:

```
library(effsize)
cohen.d()
```

Pearson product-moment correlation:

```
cor.test()
```

Standard deviation with 95% confidence limits:

```
library(Ecfun)
sd()
confint.sd()
```

Bland-Altman analysis:

```
library(blandr)
blandr.statistics()
```

Deming regression:

```
library(mcr)
mcreg()
```
